# The COVID-19 outbreak and corporate cash-holding levels: Evidence from China

**DOI:** 10.3389/fpsyg.2022.942210

**Published:** 2022-10-17

**Authors:** Donghua Zhou, Hua Zhou, Min Bai, Yafeng Qin

**Affiliations:** ^1^Department of Accountancy, Jiangxi University of Finance and Economics, Nanchang, Jiangxi, China; ^2^Waikato Management School, University of Waikato, Hamilton, Waikato, New Zealand; ^3^School of Economics and Finance, Massey University, Wellington, Manawatu Wanganui, New Zealand

**Keywords:** COVID-19 outbreak, cash-holdings level, corporate governance, management tone, China

## Abstract

By employing data from Shanghai and Shenzhen A-share markets for the period of 2019–2020, this paper examines the relationship between the degree of the COVID-19 pandemic’s impact on firms’ cash-holdings levels in China. We find that firms that are severely affected by the COVID-19 pandemic have higher current cash holdings levels, suggesting that the more positive (negative) the management tone in responding to the COVID-19 pandemic impact, the lower (higher) the firm’s current cash holdings. However, future corporate cash holdings decrease considerably irrespective of the corporate sentiment towards COVID-19. The positive sentiment of each firm’s management team towards the supply chain and the government policies results in a relative reduction of current cash holdings, whereas the severe impact on operating performance, especially the impact of the outbreak on the supply chain, demand, production and operations, and government policies, reduces the firm’ s future cash holdings. In addition, the impact of the pandemic has increased the current cash holdings of state-owned enterprises and reduced the future cash holdings of non-state-owned enterprises. Meanwhile, companies located in a city with a higher density of population or companies that experience relatively higher competition in the industry tend to undergo a severer impact on their current and future cash holdings due to the COVID-19 pandemic. Overall, this study sheds the light on stimulating the vitality of enterprise investment and promoting the domestic economic cycle.

## Introduction

The new coronavirus has been spreading rapidly since the first known infections were reported in Wuhan, China in late December 2019. On 11th March 2020, the WHO officially declared it a “global pandemic” considering the rapid spread and severity of the outbreak around the world. This global pandemic has caused an unprecedented impact on the development of the economy and society worldwide (Xi, 2020). As a response, many countries have urgently adopted a series of measures to constrain and eliminate the virus including the practice of home isolation regulation, enforcement of city lockdown, and implementation of travel restrictions. It is palpable that this outbreak has not only caused disruptions to the global manufacturing and supply chain around the world but also hampered the domestic economic cycle of countries such as China.

Any sort of pandemic adversely affects corporate profitability (globally), decreases the survival rate of organizations (Stubbart and Knight, 2016), and adversely increases uncertainty for businesses. In this turbulence managers thrive to maintain firm performance with the help of resources, especially cash holdings can be the best cushion, cash holdings are the efficient operations of a firm (Davis and Stout, 1992; Greenley and Oktemgil, 1998). And if firms have growth opportunities but do not have sufficient cash will lead to fewer opportunities, ultimately firm pass over this opportunity to their competitors at the same time firm face financing issues and does not meet current financial needs.

On the macroeconomic level, it is evident that the domestic growth rate in China has declined while the unemployment rate has increased; on the microeconomic level, the COVID-19 pandemic has decreased the demand for services and goods produced by local companies, resulting in overcapacity and reduced efficiency of the operations. Some of these companies are facing challenging times and are on the brink of insolvency. Besides these challenges, the shortage of corporates’ cash flow is another critical issue (Xiao et al., 2020). A survey on the impact of the COVID-19 pandemic on medium and large enterprises in China has found that among 212 interviewed Chinese well-known domestic firms, 23.11% of the enterprises were pessimistic, believing that their cash holdings would be consumed within 3 months. Small and medium-sized enterprises are in an even worse position in terms of the cash flow shortage. Research by Zhu et al. (2020) found that 34% of enterprises can only maintain sufficient cash to support 1 month of operations, 33.1% for 2 months, 17.91% for 3 months, and only 9.96% can last for more than 6 months.

In fact, the statement “cash is king” has been recognized by many accounting scholars (Keynes, 1937; Duchin et al., 2010; Subramaniam et al., 2011), and this view is especially salient during economic recessions. Maintaining a reasonable level of cash holdings can help companies reduce capital costs (Wang et al., 2014; Yu et al., 2019), seize investment opportunities (Wang, 2009), promote corporate innovation (Liu et al., 2017), enhance product market competitiveness (Yang et al., 2015), increase corporate value (Xiao et al., 2020) and have an important impact on the persistence and development of enterprises (Baum et al., 2006). So, to what extent has the most severe public health event since the 1918 flu (Barrios and Hochberg, 2020) affected the level of cash holdings of Chinese companies? Facing the COVID-19 outbreak impact, will the optimism or pessimism of different companies have different effects on the level of cash holdings? Furthermore, what specific challenges have caused a greater impact on these companies? Are there heterogeneity effects among different firms? These questions will be addressed in this study.

This paper uses a data sample of Shanghai and Shenzhen A-share listed companies in China from 2019 to 2020, constructing firm-level impact indicators of the COVID-19 pandemic based on textual analysis to empirically examine the impact of the new coronavirus outbreak on corporates’ cash holdings. We have found that: (1) For companies with a higher degree of impact from the COVID-19 pandemic, their current cash holdings increased for precautionary motives while their future cash holdings dropped significantly after a severe impact on their operating performance; (2) When the companies’ management team is optimistic (pessimistic) in the face of the pandemic, the current level of cash holdings of the company will significantly decrease (increase); (3) The positive sentiment of the companies’ management teams towards the supply chain logistics and the government response to the pandemic has derived a lower precautionary motive of such companies to a certain extent, which has resulted in a reduction of their current cash holdings level. However, the pandemic is likely to have a major impact on the companies’ performance due to supply chain disruption, reduction in demand, negative influence on production operations and the implementation of relevant government response policies, resulting in a decline in corporates’ cash holdings in the future; and (4) The impact of the pandemic has caused the state-owned enterprises in China to increase their current cash holdings, while private enterprises tend to reduce their future cash holdings. At the same time, the impact of the pandemic on corporate cash holdings is only significant if the company is located in a densely populated area or operates in a highly competitive industry.

Contributions of this paper are mainly as follows: First, unlike the relevant literature that examines the ability of firms’ past internal liquidity to cope with the COVID-19 outbreak (De Vito and Gomez, 2020), this paper focuses on the impact of the COVID-19 on the level of firms’ cash holdings during the epidemic, which helps to reveal the importance of firms’ precautionary cash holdings. Second, compared to the literature related to the external environment including government policies (tax extensions, bridging loans, etc.) and credit agency ratings (Acharya and Steffen, 2020), which influence firms’ fund raising and thus get through the epidemic crisis in general, this paper looks at the six relevant dimensions faced by companies themselves, such as supply chain, demand, production and operations, funding, cost, and government. This paper provides empirical evidence on the main dilemmas faced by enterprises in the COVID-19 epidemic, which offers a hand to relevant policy makers to help enterprises tide over the cash flow dilemma in a more targeted manner. Third, China had a relatively early outbreak of the COVID-19 epidemic, but controlled its spread in a very short time. Using Chinese enterprise data to study the specific mechanism and context of the impact of the COVID-19 on the level of corporate cash holdings can help provide lessons for other countries where the epidemic is still not effectively controlled, thus further promoting the global economic cycle and recovery.

## Literature review and hypotheses development

### Literature review

#### Research on COVID-19

Studies on the impact of COVID-19 outbreak are mainly focused on the mechanism of the pandemic (Altig et al., 2020; Carlsson-Szlezak et al., 2020; Guerrieri et al., 2020), macroeconomic impact (Ludvigson et al., 2020; Yang et al., 2020; Cai et al., 2021), microeconomic influence (Chen Y. et al., 2020; Hassan et al., 2020; Ding et al., 2021; Meng et al., 2021) and related policies (Fowler et al., 2020; Liu and Lu, 2020; Ou and Jia, 2020; Tang et al., 2020; Baccini et al., 2021; Durante et al., 2021; Shan et al., 2021). Carlsson-Szlezak et al. (2020) found that a health crisis can lead to households’ wealth reduction and consumer confidence decrease followed by consumption decline, resulting in insufficient demand in the market. Moreover, the increasing unemployment rate halted the production cycle and disrupted the supply chain, significantly impacting the market supply.

On the macroeconomic level, the simultaneous impact on demand and supply has nearly ruptured the global supply chain and the world economy has suffered substantial losses. According to the study by Ludvigson et al. (2020), industrial production in the United States has dropped by 12.75%, the employment rate in the service sector has dropped 17% and the number of inbound and outbound flights has decreased significantly; the severity of the impact in the macro-economy has been uncertain for the past 5 months and remains unknown. In China, the risk profile of the financial, real estate, information technology, and daily consumption industries has increased significantly while health care, utilities, and industry have become the main risk takers (Yang et al., 2020).

From the microeconomic point of view, the COVID-19 impact has led to greater negative sentiment and lower stock returns for companies (Chen Y. et al., 2020; Hassan et al., 2020). Meng et al. (2021) point out that Employee Share Schemes established on consumer altruism and employees’ sense of pride, can improve civic behavior and the internal and external perception of the company as a Corporate Social Responsible organisation, thus alleviating the negative impact of the pandemic on the enterprise. In addition to the corporations’ strategies to strive during these uncertain times, the government has also introduced a series of policies to hedge the impact of the pandemic. Compared with foreign studies which have focused on the influencing factors and effects of government policies such as home isolation and remote working (Fowler et al., 2020; Liu and Lu, 2020; Baccini et al., 2021; Durante et al., 2021), researches in China are more interested in the recovery of the domestic economy. Researchers have proposed that China should rely on the digital economy (Ou and Jia, 2020; Shan et al., 2021), to smooth the domestic circulation, and promote international and domestic double circulation (Tang et al., 2020), seeking a long-term healthy and stable development of the economy.

In addition, corporate cash holdings have been studied with firm performance in the financial context widely. For example, (Bromiley 1991; Nohria and Gulati (1996); Wan and Yiu 2009; Paeleman and Vanacker 2015; Shahzad et al., 2016; Wang et al., 2016) provide new research avenues and researchers of cash holdings indulge themselves to detect the relation of cash holdings with other organization variables. The study of Carnes et al. (2019) investigates the mediating role of slack resources between market competition and firm performance. Besides, cash holdings are also studied in relation to other variables like firm strategies, innovation, new venture performance, and research & development. On one hand, there is a mechanism necessary to facilitate the use of cash. Precise financial planning is required for the management of cash holding levels, especially from the point of view of cash flow, capital structure, and risk. On the other hand, corporate cash holdings especially are linked with financial flexibility. We believe there is a visible gap in cash allocation strategies from the financial point of view.

#### Research on influencing factors of cash holdings

The research on the influencing factors of cash holdings is divided into internal and external influencing aspects. Internal influencing factors mainly refer to corporate governance including ownership structure (Dittmar and Mahrt-Smith, 2007; Dou and Lu, 2016; Yang and Yin, 2018), executive characteristics (Kuan et al., 2012; Zhou et al., 2021) and board features (Al-Najjar and Clark, 2017; Chen RR. et al., 2020). External factors include litigation risk (Wang and Wang, 2018), legal system environment (Cui et al., 2018; Qian et al., 2019), product markets (Haushalter et al., 2007), industry growth (Yang et al., 2016), political uncertainty (Jiang et al., 2015; Xu et al., 2016; Yu et al., 2019) and macroeconomic policies (Lu and Han, 2013; Demir and Ersan, 2017; Su et al., 2020; Liu et al., 2021). As a portion of a company’s assets, cash is more likely to be disposed or misappropriated. Firms, in order to mitigate the principal-agent problem, tend to reduce their cash holdings. This strategy is often implemented in firms with stronger corporate governance (Chen RR. et al., 2020). At the same time, changes in the macroeconomic environment will also have a significant impact on corporate cash holdings since it is highly sensitive, flexible and resilient to such an environment (Rao and Zhang, 2015). In the face of macro-economic uncertainty, companies tend to increase cash holdings for speculative motives (Lu and Han, 2013) and precautionary motives (Phan et al., 2019; Zeng et al., 2020). Based on the Lehman events, Smietanka et al. (2018) found that even after controlling the volatility of investment opportunities, sales growth and the company’s shares, UK based companies did not increase investment due to record low interest rates but instead held more cash to deal with great uncertain times. Based on the impact of the COVID-19 pandemic, Xiao et al. (2020) found that the greater the impact on a company of the coronavirus pandemic, the higher the preventive value of its cash holdings, especially when the cash flow pressure is high and the external financing environment is poor.

In addition, many studies have been conducted on the impact of significant losses caused by the COVID-19 pandemic, however, most studies are using either the number of confirmed cases or deaths in the company’s location as the degree of the impact to the company. Few studies have conducted quantitative research and analysis on the impact of the COVID-19 pandemic at the individual level of the company.

With the help of the word pattern-based method, Hassan et al. (2020) constructed an indicator of the COVID-19 impact on individual companies based on the quarterly earnings conference calls. In terms of sentiment and risk, they conducted a statistical analysis of regional and industry differences in more than 80 countries including the United States; they also conducted empirical tests on the stock returns of a number of companies based on the impact of the pandemic, achieving a further refinement of the research related to the COVID-19 impact.

Unlike other countries, China is currently under a “dual circulation” development pattern in which domestic economic cycle plays a leading role while international economic cycle remains its extension and supplement. The success of this strategy will have a significant impact on China’s economic recovery and could influence the investment motivation of companies with the aim of unlocking links of production, distribution and logistics, internal consumption and more importantly to debottleneck the domestic economic cycle. Cash, as the “blood” of an enterprise, is one of the key factors affecting their investment decisions. However, the existing literature does not provide empirical evidence on how the impact of the COVID-19 pandemic affects the level of corporate cash holdings. Consequently, this study will further enhance the research on the economic consequences of the COVID-19 pandemic and the factors affecting the level of corporate cash holdings while also providing a better understanding of the obstacles in China’s domestic economic cycle recovery as well as ideas for accelerating economic recovery and development.

### Theoretical analysis and research assumptions

Enterprises always operate under a certain macroeconomic environment and their cash holdings are deemed to be affected by such environment and its policies (Yu et al., 2019). In the face of macroeconomic and policy uncertainty, companies tend to hold optimal levels of cash due to transactional motives when the economic environment is positive whereas, in times of economic recessions, these firms are inclined to hold additional cash for precautionary motives (Almeida et al., 2004). Under the negative impact of the COVID-19 pandemic, enterprises have been affected in the aspects of supply and demand, production and operations, capital, cost and government policies to a different extent. These adverse situations have further influenced the companies’ decisions regard to cash holdings levels.

Suppliers and enterprises are a community of interests and the impact of supply chain enterprises will also affect business decisions to a large extent (Di et al., 2020). Therefore, although companies themselves may be relatively less affected by the pandemic, they are likely to adjust their business decisions due to the uncertainty of supply chain companies’ operations and development. In theory, based on concerns about supply chain disruptions, companies would increase their cash holdings to hedge risks in upstream raw material procurement and downstream final product supply. In addition to the supply issue, the pandemic has also had a severe impact on the demand side. The downturn of the entire social economy directly leads to a decline in consumer spending ability and a lack of consumer confidence, which ultimately leads to a market demand decline. In this scenario, it may be better for companies to postpone their investment which will result in a decline of corporate capital investment and an increase in cash holdings (Liu et al., 2016).

At the same time, many companies have been forced to suspend production or even closure of their business since the coronavirus has been proven to be transmitted by direct means such as sneezing, coughing, droplets, inhalation at close range or by breathing the aerosols formed in the air by the droplets, contact deposition, droplets spread on the surface of objects and other indirect methods. As a consequence, the flow of goods and personnel have been restricted, production and operations halted, key business activities have been delayed and operating cash flow conditions continued to deteriorate.

In order to complete essential projects on schedule as much as possible and alleviate financial difficulties, enterprises need to take measures such as destocking and deferral of payments in order to speed up the return of funds (Xiao et al., 2020), and increase cash holdings to meet the needs of strategic adjustment and get out of production and operation difficulties as soon as possible. In addition, under the pressure of potential increase of costs due to the pandemic of key elements such as raw materials, employee cost, loan repayments and rental expenses, many small and medium-sized enterprises are forced to reduce staff and implement pay cut schemes on employees to achieve an overall cost reduction leveraged on an unenthusiastic approach, as large enterprises believe that the impact of the pandemic is just temporary.

Under the pressure of fixed cost, firms will undoubtedly increase their cash holdings in order to cope with the many uncertainties brought by the COVID-19 pandemic independently of the implementation of salary reduction schemes. While companies are actively taking a number of measures to mitigate the impact of the pandemic, the government has also been active in this extent. In the first attempts to control the fast spread of the virus, the Chinese government has implemented quarantine and closed contact management guidelines to limit the flow of people and materials, that ultimately resulted in companies reducing their operating cash flow and a reduction in corporate cash holdings; however, in the middle and later stages of the pandemic, the government issued financial support, tax concessions, recommencement of work and production support, services optimization, cost reduction and other related policies that have helped companies resume normal operations. This increase in confidence has allowed such companies to increase their cash holdings levels. The fast-changing environment requires firms to use cash in an effective way. And an optimal cash holding enables firms to tackle a pandemic, like COVID-19 efficiently. Theoretically, Deb et al. (2017) suggests that more cash holding can add more value for firms. In this study, we examine the impact of COVID-19 on firms’ cash holding level in the time of crisis.

The impact of COVID-19 on cash holding level can be endogenous to economic situations. For example, during the global financial crisis, higher cash holding firms had higher profitability, growth, sale, and raised even more debt. Recent studies also confirm the insurance role of cash holing during the COVID-19 pandemic. In general, the recent studies highlight the significance of a good cash holding level in maintaining firm value and lowering risk during negative shocks. It implies that in the COVID-19 health crisis, the role of cash holding in determining firm value can be even more pronounced.

In summary, due to the impact of the COVID-19 on the supply chain, demand, production and operation, capital, cost, government and other aspects, companies are likely to increase cash holdings for precautionary motives, however, the premise for companies to do so is that they still have reserve cash. In fact, the impact of the new coronavirus pandemic is so significant that it is likely to cause a serious decline in business performance. Firms cannot even increase the cash holdings by the means of supplementary financing and reduced investment which will lead to a passive reduction in the company’s cash holdings. Based on this, we put forward the research hypothesis of this paper:

*H1a*: Given other things being equal, firms that are hit harder by the COVID-19 pandemic will see their levels of cash holdings rise significantly due to precautionary motives.

*H1b*: Given other things being equal, the level of cash holdings of companies that are hit harder by the COVID-19 pandemic will drop significantly due to a severe decline in operating performance.

## Research design

### Data and sample

The sample consists of Shanghai and Shenzhen A-share listed companies from 2019 to 2020 in China. The COVID-19 pandemic impact of companies is constructed based on the text analysis. Cash holdings level and other data are sourced from Wind Database and China Stock Market & Accounting Research Database. This paper conducts the following screening procedures for the initial sample: (1) Eliminate listed companies in the financial and insurance industries; (2) Eliminate listed companies with missing financial data; (3) Eliminate ST and *ST companies with abnormal financial conditions, whose ability to continue as a going concern is affected and the data will be more volatile. After completing such screening, we have a total of 7,133 company-year observations. In order to eliminate the influence of outliers, this paper carried out the upper and lower 1% quantiles for all continuous variables.

### Model and variable definition

With reference to Wang et al. (2020), Yang and Yin (2018), Qian et al. (2019) and other relevant literature on factors affecting cash holdings, this model includes not only control variables of corporate financial characteristics, corporate governance features and macroeconomic variables but also Industry, Year and Province effects. At the same time, considering the differences in the focus of the discussion in the performance review and future outlook sections of the MD&A and their information content reflecting different periods (Bryan, 1997; Li, 2010; Meng et al., 2017), this paper examines the impact of the extent to which the firm is affected by the outbreak in the current period (i.e., the performance review section) on the firm’s current cash holdings and the extent to which the firm expects to be affected by the outbreak (i.e., the future outlook section) on the firm’s future cash holdings. The specific models are as follows:


(1) $$\begin{array}{c} {\mathrm{Cash}}_{i,t}=\alpha_{0}+\alpha_{1}\mathrm{Covid}1_{i,t}+\sum \mathrm{Controls}\\ +\sum \mathrm{Year}+\sum \mathrm{Industry}+\sum \mathrm{Province}+\varepsilon \end{array}$$

(2) $$\begin{array}{c} {\mathrm{Cash}}_{i,t+1}=\beta_{0}+\beta_{1}\mathrm{Covid}2_{i,t}+\sum \mathrm{Controls}+\sum \mathrm{Year}\\ +\sum \mathrm{Industry}+\sum \mathrm{Province}+\varepsilon \end{array}$$

Among them, cash holdings (Cash) with reference to Yang and Yin (2018), are measured by dividing the sum of monetary funds and trading financial assets by net assets. Moreover, net assets are the balance of total assets minus cash and cash equivalents (net assets in this study will be denoted as per the aforementioned definition).

Since the impact of the COVID-19 epidemic on firms cannot be directly measured accurately, we did not use the number of cases or deaths of the epidemic in the place where the firm located as our main explanatory variable which does not capture the differences in the impact of the epidemic on different firms in the same region (although we also run a robustness test using these indicators). Instead, referring to Hassan et al. (2020), the level of impact of the COVID-19 pandemic is defined as follows: In order to distinguish the different effects of the performance review and the future outlook, this paper refines the impact degree of the pandemic by further constructing Covid1 and Covid2 indicators; we use the number of times the performance review section mention the COVID-19 pandemic divided by the total number of sentences in the performance review section to measure Covid1 and use the number of times the future outlook section mention the COVID-19 pandemic divided by the total number of sentences in the future outlook section to measure Covid2.

The control variables include: (1) Enterprise Size (Size), measured by the logarithm of the company’s total assets at the end of the year; (2) Financial leverage (Lev), measured by dividing the company’s total liabilities by the company’s total assets; (3) Cashflow, measured by dividing cash flow from operating activities by total assets; (4) Net working capital (NWC), measured by dividing net working capital by net assets, where net working capital is the working capital remaining after deducting cash and cash equivalents; (5) Bank debt (Bankdebt), measured by the sum of short-term borrowings and long-term borrowings divided by total liabilities; (6) Capital expenditure (Capex), measured by dividing capital expenditure by total assets; (7) Dividend, defined as a dummy variable that has assigned a value of 1 when the company distributes cash dividends in the current year, otherwise it is assigned a value of 0; (8) Property rights (Soe), which assigns a value of 1 if the company’s property rights are state-owned and in any other case it assigns a value of 0; (9) Board size (Board), measured by the total number of board members of the company; (10) Enterprise age (Age), measured by the logarithm of the listing year plus 1; (11) Investment opportunities (Tobinq), defined as total market capitalization divided by the total assets; (12) Growth, measured by the growth rate of operating income; (13) GDP growth rate (GDPg), measured by the city-level GDP growth rate where the company is registered.

## Results and analysis

### Variable descriptive statistics

Panel A in Table 1 lists the descriptive statistics for the main variables. The mean and median of cash holdings (Cash) have a value of 0.318 and 0.220 respectively; the maximum and minimum values are 1.741 and 0.016 indicating that under the exogenous impact of the COVID-19 pandemic, the cash holdings of different listed companies are different which is beneficial to the regression analysis in this study. In addition, these values are a little smaller than Hu et al. (2019), whose sample period is 2001–2014, which is consistent with our theoretical analysis that the epidemic had a large impact on firms’ operating activities, which overall caused a slight decrease in cash holdings. The mean values of Covid1 and Covid2 are 0.076 and 0.057 respectively, indicating that on average 7.6 (5.7) out of 100 sentences are related to the COVID-19 pandemic in the performance review (future outlook) section. This shows that the management team of the sample companies believes that the COVID-19 pandemic has had a greater impact on the companies’ operating performance in the current year and will also have an impact on the companies’ future development. The descriptive statistics of other control variables are basically consistent with the previous literature and some variables (e.g., Tobinq) have a certain degree of maximum value. In order to prevent the influence of extreme values on the regression results, the upper and lower 1% quantiles were abbreviated for all continuous variables.

**Table 1:** Descriptive statistics of each variable and its mean difference.

Variable	*N*	Mean	SD	Min	p25	p50	p75	Max
**Panel A: Descriptive statistics**								
Cash	7,133	0.318	0.303	0.016	0.121	0.220	0.406	1.741
Covid1	7,133	0.076	0.105	0.000	0.000	0.017	0.128	0.453
Covid2	7,133	0.057	0.084	0.000	0.000	0.016	0.088	0.394
Size	7,133	22.309	1.304	20.025	21.359	22.095	23.032	26.398
Lev	7,133	0.410	0.199	0.058	0.250	0.405	0.555	0.884
Cashflow	7,133	0.060	0.064	−0.139	0.022	0.058	0.096	0.252
NWC	7,133	0.036	0.232	−0.621	−0.106	0.039	0.185	0.584
Bankdebt	7,133	0.252	0.211	0.000	0.041	0.229	0.414	0.759
Capex	7,133	0.045	0.043	0.000	0.014	0.032	0.062	0.207
Dividend	7,133	0.622	0.485	0.000	0.000	1.000	1.000	1.000
Soe	7,133	0.302	0.459	0.000	0.000	0.000	1.000	1.000
Board	7,133	8.368	1.591	5.000	7.000	9.000	9.000	14.000
Age	7,133	3.033	0.283	2.197	2.890	3.045	3.219	3.664
Tobinq	7,133	2.450	1.912	0.805	1.258	1.831	2.848	11.630
Growth	7,133	0.054	0.286	−0.994	−0.061	0.062	0.180	1.111
GDPg	7,133	0.049	0.064	−0.250	0.022	0.048	0.074	0.251
Variables	Mean			Diff (1)–(2)	Mean			Diff (4)–(5)
	Covid1 = 0 (*N* = 3,114)	Covid1 > 0 (*N* = 4,019)			Covid2 = 0 (*N* = 1,490)	Covid2 > 0 (*N* = 1,837)		
**Panel B: Comparative analysis 2**								
Cash	0.292	0.338		−0.046^***^	0.309	0.283		0.026^***^
Size	22.263	22.345		−0.082^***^	22.325	22.469		−0.144^***^
Lev	0.415	0.407		0.007	0.416	0.432		−0.016^**^
Cashflow	0.060	0.060		0.000	0.059	0.060		−0.001
NWC	0.035	0.037		−0.002	0.037	0.007		0.029^***^
Bankdebt	0.260	0.245		0.015^***^	0.259	0.255		0.004
Capex	0.045	0.045		0.000	0.045	0.043		0.002^*^
Dividend	0.730	0.538		0.193^***^	0.515	0.458		0.057^***^
Soe	0.307	0.298		0.009	0.281	0.353		−0.073^***^
Board	8.366	8.369		−0.003	8.331	8.415		−0.084
Age	3.026	3.039		−0.013^**^	3.043	3.094		−0.051^***^
Tobinq	2.320	2.551		−0.232^***^	2.613	2.258		0.355^***^
Growth	0.080	0.035		0.045^***^	0.055	0.003		0.052^***^
GDPg	0.067	0.034		0.033^***^	0.031	0.031		0.000

***, **, and * indicate statistical significance at the 1%, 5%, and 10% levels, respectively.

Panel B in Table 1, columns (1) and (2), shows the comparison results of the mean value of each variable grouped by whether it has been affected by the COVID-19 pandemic (Covid1). The values difference between columns (1) and (2) is presented in column (3) and it indicates that in the performance review section, the mean value of cash holdings (Cash) of the sample impacted by the COVID-19 (Covid1 > 0) is significantly higher than the mean value of the sample not affected by the COVID-19 (Covid1 = 0). Additionally, columns (4) and (5) shows the mean value of each variable grouped by Covid2, column (6) shows that in the future projections, the mean value of cash holdings impacted by the COVID-19 (Covid2 > 0) is significantly lower. It demonstrates that the past and future impact of the COVID-19 pandemic will have different effects on corporate cash holdings levels, which lays the foundation for subsequent regression analysis.

### Empirical regression results

This study implements the ordinary least square (OLS) model to empirically test the COVID-19 pandemic impact on corporate cash holdings (results are shown in Table 2). The regression results in Table 2 indicate that the coefficient on constraint on the degree of the COVID-19 pandemic impact is 0.075 in their performance review (with a standard error of 0.105), and −0.124 in the future projections (with a standard error of 0.084). This implies that a one-standard-deviation increase in the degree to which a company’s current operations have been affected by the COVID-19 pandemic, its cash holdings have increased by 2.60% (0.105 × 0.075/0.303), while for every standard deviation increase in the extent to which a company expects its future operations to be affected by the pandemic, its cash holdings decrease by 3.44% (0.084 × 0.124/0.303). Furthermore, this study undertakes an additional regression by using Bankdebt, Capex and Roa as dependent variables (result is shown in Table 3). Column 1 to Column 3 in Table 3 indicate a positive relationship between the degree of the COVID-19 pandemic impact and Bankdebt, a negative relationship with Capex and no significant relationship with Roa. These results indicate that firms affected by the pandemic will indeed increase their cash holdings by the means of additional financing and reducing investment due to precautionary motives. Columns (4) to (6) in Table 3 show that in the future, listed companies that are expected to be impacted by the COVID-19 pandemic, have no significant relationship with bank debt (Bankdebt) and capital expenditure (Capex), but instead have a significant negative correlation with return on total assets (Roa) at the statistical level and the economic sense (A one-standard-deviation increase in Covid3 leads to approximately a 3.05%^1^ decrease in Roa) This indicates that companies expected to be impacted by the pandemic will be forced to reduce their cash holdings due to the severe detriment on their operating performance.

**Table 2:** COVID-19 impact and corporate cash holdings.

	(1)	(2)
	Cash*_t_*	Cash*_t_*_ + 1_
Covid1	0.075^*^ (1.95)	
Covid2		−0.124^***^ (−2.62)
Size	−0.009^***^ (−2.59)	−0.008^**^ (−2.27)
Lev	−0.647^***^ (−21.79)	−0.593^***^ (−18.55)
Cashflow	0.171^***^ (3.18)	0.219^***^ (3.41)
NWC	−0.390^***^ (−14.97)	−0.397^***^ (−14.52)
Bankdebt	−0.309^***^ (−17.60)	−0.292^***^ (−14.95)
Capex	−1.071^***^ (−13.06)	−0.861^***^ (−9.22)
Dividend	0.062^***^ (9.17)	0.044^***^ (5.59)
Soe	0.015^*^ (1.70)	0.016^*^ (1.81)
Board	0.003 (1.22)	0.001 (0.43)
Age	−0.142^***^ (−9.88)	−0.103^***^ (−6.53)
Tobinq	0.025^***^ (8.59)	0.016^***^ (5.50)
Growth	0.020^*^ (1.79)	0.016 (1.04)
GDPg	0.100^**^ (2.25)	0.411^***^ (2.88)
_cons	1.204^***^ (12.93)	1.074^***^ (11.16)
*N*	7,133	3,327
Year	Y	Y
Industry	Y	Y
Province	Y	Y
Adjust *R*^2^	0.427	0.415

***, **, * indicate statistical significance at the 1%, 5%, and 10% levels, respectively; the *t* value is in brackets; the regression in this table is clustered according to the company code.

**Table 3:** The COVID-19 impact and financing, investment, and total asset return.

	(1)	(2)	(3)	(4)	(5)	(6)
	Bankdebt*_t_*	Capex*_t_*	ROA*_t_*	Bankdebt*_t_*_ + 1_	Capex*_t_*_ + 1_	ROA*_t_*_ + 1_
Covid1	0.066^**^ (2.38)	−0.017^***^ (−2.76)	0.080 (0.09)			
Covid2				−0.002 (−0.04)	−0.012 (−1.41)	−3.542^**^ (−2.21)
Size	0.001 (0.48)	0.001 (1.44)	1.131^***^ (14.46)	−0.001 (−0.18)	0.001^**^ (2.15)	1.243^***^ (12.16)
Lev	0.413^***^ (20.12)	−0.012^***^ (−2.85)	−8.840^***^ (−11.94)	0.409^***^ (17.70)	−0.015^***^ (−2.95)	−8.402^***^ (−8.43)
Cashflow	−0.435^***^ (−10.52)	0.064^***^ (7.43)	32.864^***^ (19.06)	−0.364^***^ (−6.57)	0.047^***^ (4.12)	34.624^***^ (15.43)
NWC	0.012 (0.78)	−0.026^***^ (−8.49)	2.596^***^ (5.16)	0.024 (1.30)	−0.026^***^ (−7.16)	2.217^***^ (3.26)
Bankdebt		0.035^***^ (10.98)	−0.133 (−0.30)		0.037^***^ (9.79)	−0.045 (−0.07)
Capex	0.726^***^ (11.31)		2.957 (1.62)	0.782^***^ (10.09)		3.113 (1.33)
Dividend	−0.028^***^ (−5.32)	0.009^***^ (8.03)	3.911^***^ (22.61)	−0.027^***^ (−4.06)	0.008^***^ (5.51)	2.664^***^ (13.48)
Soe	−0.025^***^ (−3.62)	−0.007^***^ (−4.87)	−0.077 (−0.40)	−0.024^***^ (−3.16)	−0.008^***^ (−4.88)	−0.126 (−0.52)
Board	0.001 (0.34)	−0.000 (−0.21)	−0.050 (−0.97)	0.000 (0.23)	−0.000 (−0.08)	−0.021 (−0.31)
Age	0.002 (0.17)	−0.014^***^ (−6.27)	0.476 (1.62)	0.000 (0.03)	−0.016^***^ (−5.36)	0.352 (0.90)
Tobinq	−0.012^***^ (−8.28)	0.002^***^ (4.64)	0.768^***^ (13.10)	−0.012^***^ (−7.08)	0.002^***^ (4.50)	0.710^***^ (10.93)
Growth	−0.014 (−1.60)	0.010^***^ (5.06)	7.732^***^ (21.74)	−0.021^*^ (−1.87)	0.013^***^ (5.23)	7.315^***^ (15.84)
GDPg	−0.013 (−0.36)	0.003 (0.38)	1.883 (1.35)	−0.154 (−1.13)	0.002 (0.06)	3.737 (1.09)
_cons	0.187^***^ (2.58)	0.068^***^ (4.12)	−25.869^***^ (−12.99)	0.238^***^ (2.94)	0.068^***^ (3.45)	−27.294^***^ (−10.23)
*N*	7,133	7,133	7,133	3,327	3,327	3,327
Year	Y	Y	Y	Y	Y	Y
Industry	Y	Y	Y	Y	Y	Y
Province	Y	Y	Y	Y	Y	Y
Adjust *R*^2^	0.334	0.214	0.488	0.321	0.233	0.487

***, **, * indicate statistical significance at the 1%, 5%, and 10% levels, respectively; the *t* value is in brackets; the regression in this table is clustered according to the company code.

In conclusion, the COVID-19 pandemic impact on the level of corporate cash holdings cannot be generalized. Specially in the performance review section, the extent of the impact of the pandemic on companies is positively correlated with their cash holdings, which proves the research hypothesis H1a of this study, whereas in the future projections, the extent of the impact of the pandemic on companies is negatively correlated with their cash holdings, which verifies the H1b hypothesis.

### Robustness test

#### COVID-19 impact and corporate cash holdings: A measure of changing independent variables

In order to reduce the bias caused by the impact of the COVID-19 pandemic (*Covid*), this paper uses the cumulative number of confirmed cases (Case, as of April 30 at year *t* + 1, taking the logarithm of the cumulative number of confirmed cases in the city where the company is registered plus one), cumulative deaths (Death, as of April 30 at year of *t* + 1, taking the logarithm of the cumulative number of deaths in the city where the company is registered plus one) as the independent variables of the regression. Results are shown in Table 4 and it indicates that the cumulative confirmed cases (Case) and cumulative deaths (Death) are significantly positively correlated with the current cash holding level of the company at the level of 1% and 5%, respectively. The results are consistent with the afore-mentioned conclusions, indicating that the regression results in this paper are robust.

**Table 4:** COVID-19 impact and corporate cash holdings: a measure of changing independent variables.

	(1)	(2)
	Cash*_t_*	Cash*_t_*
Case	0.010^***^ (3.10)	
Death		0.012^**^ (2.33)
Size	−0.009^**^ (−2.53)	−0.009^**^ (−2.49)
Lev	−0.652^***^ (−21.77)	−0.650^***^ (−21.71)
Cashflow	0.182^***^ (3.36)	0.180^***^ (3.32)
NWC	−0.393^***^ (−15.01)	−0.393^***^ (−14.98)
Bankdebt	−0.308^***^ (−17.48)	−0.309^***^ (−17.60)
Capex	−1.064^***^ (−12.90)	−1.064^***^ (−12.88)
Dividend	0.061^***^ (9.02)	0.061^***^ (9.04)
Soe	0.012 (1.40)	0.013 (1.47)
Board	0.003 (1.24)	0.003 (1.21)
Age	−0.143^***^ (−9.92)	−0.143^***^ (−9.91)
Tobinq	0.025^***^ (8.33)	0.025^***^ (8.37)
Growth	0.017 (1.49)	0.017 (1.46)
GDPg	0.090^**^ (1.99)	0.104^**^ (2.30)
_cons	1.140^***^ (11.96)	1.177^***^ (12.46)
*N*	7,067	7,067
Year	Y	Y
Industry	Y	Y
Province	Y	Y
Adjust *R*^2^	0.428	0.428

***, **, * indicate statistical significance at the 1%, 5%, and 10% levels, respectively; the *t* value is in brackets; the regression in this table is clustered according to the company code.

#### COVID-19 impact and corporate cash holdings: A measure of changing dependent variables

In order to avoid the possible errors caused by a single cash holding measurement, we further use Cash2 (measured by dividing monetary funds by net assets) and Cash3 (measured by dividing monetary funds by total assets) as dependent variables to develop a regression model. The outcomes are shown in Table 5. These results are consistent with the main regression results, indicating that the conclusions of this paper are robust.

**Table 5:** COVID-19 impact and corporate cash holdings: a measure of changing dependent variables.

	(1)	(2)	(3)	(4)
	Cash2*_t_*	Cash3*_t_*	Cash2_*t * +1_	Cash3_*t * +1_
Covid1	0.081^**^ (2.12)	0.039^**^ (2.21)		
Covid2			−0.086^*^ (−1.86)	−0.045^*^ (−1.81)
Size	−0.013^***^ (−3.49)	−0.007^***^ (−3.90)	−0.011^***^ (−2.90)	−0.006^***^ (−3.05)
Lev	−0.402^***^ (−13.87)	−0.201^***^ (−14.21)	−0.374^***^ (−11.86)	−0.190^***^ (−12.03)
Cashflow	0.205^***^ (3.97)	0.115^***^ (4.45)	0.239^***^ (3.78)	0.150^***^ (4.44)
NWC	−0.271^***^ (−10.08)	−0.146^***^ (−12.34)	−0.281^***^ (−10.05)	−0.153^***^ (−11.79)
Bankdebt	−0.227^***^ (−13.66)	−0.126^***^ (−14.62)	−0.221^***^ (−11.89)	−0.129^***^ (−12.69)
Capex	−0.783^***^ (−9.93)	−0.389^***^ (−10.03)	−0.661^***^ (−7.36)	−0.344^***^ (−7.28)
Dividend	0.050^***^ (7.93)	0.028^***^ (9.10)	0.032^***^ (4.17)	0.021^***^ (5.18)
Soe	0.030^***^ (3.74)	0.016^***^ (4.07)	0.028^***^ (3.20)	0.014^***^ (3.11)
Board	0.004 (1.60)	0.001 (0.94)	0.002 (1.04)	0.001 (0.84)
Age	−0.103^***^ (−7.26)	−0.043^***^ (−6.67)	−0.078^***^ (−4.96)	−0.033^***^ (−4.28)
Tobinq	0.020^***^ (6.66)	0.008^***^ (6.34)	0.012^***^ (3.96)	0.005^***^ (3.28)
Growth	0.014 (1.36)	0.009^*^ (1.83)	0.017 (1.18)	0.014^**^ (1.96)
GDPg	0.083^**^ (2.03)	0.048^**^ (2.33)	0.318^**^ (2.32)	0.170^**^ (2.28)
_cons	0.952^***^ (10.40)	0.516^***^ (11.96)	0.858^***^ (9.01)	0.482^***^ (10.15)
*N*	7,133	7,133	3,327	3,327
Year	Y	Y	Y	Y
Industry	Y	Y	Y	Y
Province	Y	Y	Y	Y
Adjust *R*^2^	0.287	0.320	0.277	0.299

***, **, * indicate statistical significance at the 1%, 5%, and 10% levels, respectively; the *t* value is in brackets; the regression in this table is clustered according to the company code

#### COVID-19 impact and corporate cash holdings: Independent analysis of the years

Since the COVID-19 pandemic outbreak started in December 2019 and was constrained in 2020 in China, the impact of pandemic prevention and control measures adopted by enterprises in different regions were diverse especially for the 2019 term. Therefore, for this study, we performed regression analysis again segregating the data by year with the results shown in column (1) and column (2) of Table 6. The results show that although the relationship between the impact of COVID-19 and corporate cash holdings is positive in both years, the impact on companies in 2019 is more significant than in 2020 from both statistical and economic point of view. It shows that companies affected by the COVID-19 in 2019 have recognized the negative influence of the outbreak on their production capacity and operations, resulting in an increased cash holdings level due to the precautionary motives in order to better manage the uncertainty brought by the pandemic. The reason for a non-significant impact of the COVID-19 on the level of corporate cash holdings in 2020 is potentially due to firms not having the ability to increase their cash holdings due to the already spread outbreak and implemented lockdown policies.

**Table 6:** COVID-19 impact and corporate cash holdings: independent analysis of the years and excluding loss-making companies.

	Year = 2019	Year = 2020	Netprofit > 0	
	(1)	(2)	(3)	(4)
	Cash*_t_*	Cash*_t_*	Cash*_t_*	Cash_*t* + 1_
Covid1	3.748^***^ (3.29)	0.064 (1.61)	0.057 (1.34)	
Covid2				−0.146^***^ (−2.71)
Size	−0.011^**^ (−2.49)	−0.009^**^ (−2.32)	−0.012^***^ (−3.00)	−0.010^**^ (−2.51)
Lev	−0.528^***^ (−15.87)	−0.742^***^ (−21.32)	−0.656^***^ (−19.82)	−0.594^***^ (−15.89)
Cashflow	0.319^***^ (4.52)	0.057 (0.82)	0.174^***^ (3.00)	0.177^**^ (2.49)
NWC	−0.352^***^ (−11.61)	−0.422^***^ (−14.56)	−0.405^***^ (−14.28)	−0.410^***^ (−13.33)
Bankdebt	−0.295^***^ (−14.94)	−0.313^***^ (−14.65)	−0.316^***^ (−16.87)	−0.307^***^ (−14.06)
Capex	−1.102^***^ (−10.61)	−1.079^***^ (−11.07)	−1.131^***^ (−12.94)	−0.858^***^ (−8.45)
Dividend	0.073^***^ (8.43)	0.055^***^ (6.31)	0.055^***^ (7.71)	0.035^***^ (4.26)
Soe	0.022^**^ (2.36)	0.009 (0.98)	0.011 (1.17)	0.011 (1.14)
Board	0.003 (1.19)	0.002 (0.71)	0.002 (0.65)	−0.000 (−0.03)
Age	−0.107^***^ (−6.07)	−0.165^***^ (−10.59)	−0.145^***^ (−9.49)	−0.115^***^ (−6.82)
Tobinq	0.030^***^ (6.37)	0.022^***^ (7.09)	0.025^***^ (8.09)	0.017^***^ (5.36)
Growth	0.010 (0.61)	0.035^**^ (2.15)	0.003 (0.22)	−0.005 (−0.28)
GDPg	0.103^*^ (1.73)	0.481^***^ (3.09)	0.094^*^ (1.93)	0.466^***^ (3.02)
_cons	1.037^***^ (9.29)	1.366^***^ (13.26)	1.300^***^ (12.98)	1.191^***^ (11.40)
*N*	3,367	3,766	6,327	2,896
Year	-	-	Y	Y
Industry	Y	Y	Y	Y
Province	Y	Y	Y	Y
Adjust *R*^2^	0.399	0.445	0.425	0.411

***, **, * indicate statistical significance at the 1%, 5%, and 10% levels, respectively; the *t* value is in brackets; the regression in this table is clustered according to the company code.

#### COVID-19 impact and corporate cash holdings: Excluding loss-making companies

At the same time, we have further removed the loss-making enterprises from the regression to avoid their influence on the research conclusions. Outcomes are shown in columns (3) and (4) of Table 6. The results show that the positive relationship between the degree of impact of the COVID-19 pandemic on companies and the level of current cash holdings is no longer significant after excluding loss-making firms. The expectation that companies will be impacted by the pandemic still has a significant negative correlation with the companies’ cash holdings in the following year. It advises that the impact of the COVID-19 pandemic not only has a significant impact on the current cash holdings of loss-making companies but also has a huge impact on the future cash holdings of other companies, indicating that the conclusions of this paper are relatively stable.

### Further analysis

#### The impact of The COVID-19 pandemic on management tone and corporate cash holdings

Existing studies have found that there is a significant relationship between management tone and the future performance of enterprises (Xie and Lin, 2015). Based on the word lists created by Loughran and McDonald (2011), we have constructed a positive word list and negative word list by using Python Jieba Dataset in this paper.

We have further explored the different effects of (1) positive management tone (POS, counting the positive words appearing in sentences when the management team have mentioned the word COVID-19, including sentences before and after). We have then used this count to divide it by the total number of COVID-19 words in the MD&A. (2) Negative Management Tone (NEG, counting the negative words appearing in sentences when the management team have mentioned the word COVID-19, including sentences before and after) on corporate cash holdings. Consistent with the aforementioned analysis, in this session, we also separate the impact of different tones on corporate cash holdings based on the performance review section (POS1, NEG1) and future outlook section (POS2, NEG2). Statistical description is shown in Table 7.

**Table 7:** The impact of the COVID-19 epidemic and management tone descriptive statistics.

Variable	*N*	Mean	SD	Min	p25	p50	p75	max
POS1	7,133	1.263	1.618	0.000	0.000	0.667	2.077	7.500
NEG1	7,133	0.734	0.950	0.000	0.000	0.333	1.222	4.333
POS2	7,133	0.956	1.610	0.000	0.000	0.000	1.333	9.000
NEG2	7,133	0.837	1.225	0.000	0.000	0.000	1.333	6.000

In theory, the precautionary motivation approach suggests that the more negative the management tone, the higher the level of cash holdings; however, there are also studies suggesting that if a companies’ performance declines, the more negative the management tone the lower the level of cash holdings. Table 8 shows the results of the regression analysis of the relationship between management tone and current and future corporate cash holdings level under the COVID-19 pandemic impact. Column (1) of Table 8 indicate that in the historical performance section, the management’s positive tone is significantly negatively correlated with the company’s current cash holdings, and a one-standard-deviation increase in POS1 is related to 2.67% (0.005 × 1.618/0.303) decrease in company’s current cash holdings. And column (2) shows that the negative tone is significantly positively correlated with the company’s current cash holdings, which increases 1.88% (0.006 × 0.950/0.303) with a one-standard-deviation increase in NEG1. These suggestions are in line with the aforementioned precautionary motive theory. Columns (3) and (4) of Table 8 show that in the future outlook area, whether the management tone is positive or negative, it is negatively correlated with the company’s cash holdings in the next year and the absolute value of the negative management tone coefficient is larger (0.005 × 1.610/0.262 = 3.07% vs. 0.007 × 1.225/0.262 = 3.27%). This is also consistent with the aforementioned research hypothesis that the corporate performance is severely damaged by the COVID-19 pandemic.

**Table 8:** COVID-19 impact, management tone and corporate cash holdings.

	(1) Cash*_t_*	(2) Cash*_t_*	(3) Cash_*t* +1_	(4) Cash_*t* +1_
POS1	−0.005^**^ (−2.31)			
NEG1		0.006^*^ (1.70)		
POS2			−0.005^**^ (−2.08)	
NEG2				−0.007^**^ (−2.49)
Size	−0.009^**^ (−2.52)	−0.010^***^ (−2.63)	−0.008^**^ (−2.29)	−0.008^**^ (−2.30)
Lev	−0.648^***^ (−21.82)	−0.648^***^ (−21.81)	−0.591^***^ (−18.52)	−0.591^***^ (−18.53)
Cashflow	0.175^***^ (3.26)	0.178^***^ (3.31)	0.214^***^ (3.32)	0.211^***^ (3.27)
NWC	−0.390^***^ (−14.97)	−0.391^***^ (−15.01)	−0.395^***^ (−14.42)	−0.396^***^ (−14.46)
Bankdebt	−0.309^***^ (−17.58)	−0.308^***^ (−17.57)	−0.293^***^ (−14.95)	−0.293^***^ (−14.94)
Capex	−1.078^***^ (−13.15)	−1.078^***^ (−13.15)	−0.855^***^ (−9.15)	−0.849^***^ (−9.07)
Dividend	0.062^***^ (9.17)	0.062^***^ (9.20)	0.044^***^ (5.55)	0.044^***^ (5.50)
Soe	0.015^*^ (1.74)	0.015^*^ (1.71)	0.017^*^ (1.88)	0.017^*^ (1.95)
Board	0.003 (1.24)	0.003 (1.25)	0.001 (0.46)	0.001 (0.42)
Age	−0.142^***^ (−9.86)	−0.141^***^ (−9.84)	−0.103^***^ (−6.54)	−0.103^***^ (−6.50)
Tobinq	0.025^***^ (8.52)	0.025^***^ (8.56)	0.016^***^ (5.52)	0.016^***^ (5.54)
Growth	0.020^*^ (1.77)	0.020^*^ (1.79)	0.019 (1.23)	0.018 (1.15)
GPDg	0.099^**^ (2.23)	0.100^**^ (2.25)	0.405^***^ (2.86)	0.407^***^ (2.87)
_cons	1.198^***^ (12.88)	1.202^***^ (12.89)	1.076^***^ (11.22)	1.079^***^ (11.21)
*N*	7,133	7,133	3,327	3,327
Year	Y	Y	Y	Y
Industry	Y	Y	Y	Y
Province	Y	Y	Y	Y
Adjust *R*^2^	0.427	0.427	0.415	0.415

***, **, and * indicate statistical significance at the 1%, 5%, and 10% levels, respectively; the *t* value is in brackets; the regression in this table is clustered according to the company code.

#### COVID-19 impact, corporate cash holdings and six major topics

In order to further explore the specific mechanism leading the impact of the pandemic on corporate cash holdings, this study has referred to Hassan et al. (2020)’s research, and considers the company’s supply chain (Supply), demand (Demand), production and operation (P&O), capital (Finance), cost (Cost), government (GOV) and other six major themes of sentiment on the impact of the COVID-19 pandemic on the relationship between corporate cash holdings. Table 9 is the descriptive statistics of the management’s sentiment on the six major topics in the performance review section and the future outlook section. Table 9 shows that, among the six topics, management pays relatively high attention to production and operations, demand, government, and supply chain. Specifically, in the performance review section, when talking about the impact of the epidemic on the six major topics, the number of the companies with an overall positive attitude is significantly higher than those with a negative attitude; while in the future outlook section, the number of the companies with an overall positive attitude is basically the same as those with a negative attitude, and the sample companies have increased concerns about the supply chain, finance and costs. The possible reason for this distribution is that the 2019 performance review section is mainly a discussion of the fiscal year period, while the outbreak in China only started at the end of 2019, so most of the sample’s discussion and analysis about the outbreak in that time period may not be based on the company itself being directly impacted by the outbreak, but on the industry level as a whole (e.g., competitors).

**Table 9:** COVID-19 impact, corporate cash holdings and six major topics.

	POS			NEG		
Variable	*N*	mean	p50	*N*	mean	p50
Supply1	1,346	0.191	0.147	643	0.126	0.100
Demand1	1931	0.239	0.194	786	0.177	0.140
P&O1	2,838	0.333	0.277	885	0.226	0.182
Finance1	789	0.149	0.122	530	0.118	0.093
Cost1	978	0.179	0.140	529	0.132	0.109
GOV1	1917	0.204	0.160	715	0.133	0.103
Supply2	777	0.172	0.130	935	0.133	0.103
Demand2	1,175	0.204	0.149	1,102	0.144	0.111
P&O2	1,656	0.249	0.179	1,602	0.181	0.138
Finance2	440	0.144	0.114	664	0.128	0.096
Cost2	480	0.150	0.122	493	0.123	0.103
GOV2	1,231	0.193	0.136	1,066	0.151	0.113

Table 10 shows the regression results of the six major topics of the COVID-19 pandemic and corporate cash holdings level in the performance review. Panel A in Table 10 shows that when the management tone is positive, the regression coefficients of supply chain (Supply1) and government (GOV1) are significantly negative. This suggests that companies have confidence in the supply chain and government resulting in a weakening effect of companies’ preventive motivation and a significant drop of current cash holding levels. Panel B in Table 10 shows that when the management tone is negative, the six major topics have no significant relationship with the company’s current cash holdings levels. The possible reason for this is that, as shown in Table 9, the number of sample companies showing an overall negative attitude when talking about the impact of the epidemic on the six major topics is too small.

**Table 10:** Six major topics of COVID-19 and corporate cash holdings (performance review section).

	(1)	(2)	(3)	(4)	(5)	(6)
	Cash*_t_*	Cash*_t_*	Cash*_t_*	Cash*_t_*	Cash*_t_*	Cash*_t_*
**Panel A: Positive attitude and corporate cash holdings**						
Supply1	−0.095^**^ (−2.35)					
Demand1		−0.029 (−0.90)				
P&O1			−0.019 (−1.04)			

Finance1				−0.079 (−1.03)		
Cost1					−0.081 (−1.64)	
GOV1						−0.054^*^ (−1.68)
Size	−0.005 (−0.90)	−0.014^***^ (−2.62)	−0.009^**^ (−1.97)	−0.008 (−1.13)	−0.008 (−1.14)	−0.009^*^ (−1.90)
Lev	−0.790^***^ (−12.31)	−0.752^***^ (−14.27)	−0.689^***^ (−17.03)	−0.609^***^ (−9.09)	−0.624^***^ (−9.52)	−0.640^***^ (−13.64)
Cashflow	0.069 (0.59)	0.099 (0.98)	0.154^**^ (2.00)	0.444^***^ (3.03)	0.161 (1.23)	0.087 (0.97)
NWC	−0.462^***^ (−9.43)	−0.464^***^ (−11.42)	−0.424^***^ (−13.07)	−0.379^***^ (−6.62)	−0.386^***^ (−6.92)	−0.403^***^ (−9.11)
Bankdebt	−0.312^***^ (−8.27)	−0.325^***^ (−10.48)	−0.324^***^ (−13.48)	−0.275^***^ (−6.08)	−0.277^***^ (−7.27)	−0.303^***^ (−11.13)
Capex	−1.069^***^ (−5.91)	−1.187^***^ (−7.63)	−1.072^***^ (−9.17)	−0.934^***^ (−4.03)	−0.840^***^ (−4.58)	−0.961^***^ (−7.12)
Dividend	0.054^***^ (3.62)	0.057^***^ (4.68)	0.055^***^ (5.89)	0.032^*^ (1.89)	0.042^***^ (3.15)	0.050^***^ (4.46)
Soe	0.001 (0.08)	−0.013 (−1.07)	−0.010 (−1.03)	0.030^*^ (1.71)	0.000 (0.01)	0.006 (0.56)
Board	0.003 (0.64)	0.001 (0.14)	−0.001 (−0.19)	0.000 (0.08)	0.003 (0.74)	0.001 (0.21)
Age	−0.176^***^ (−6.15)	−0.174^***^ (−7.63)	−0.153^***^ (−8.42)	−0.149^***^ (−4.86)	−0.133^***^ (−4.80)	−0.159^***^ (−6.77)
Tobinq	0.018^***^ (3.55)	0.017^***^ (4.03)	0.019^***^ (5.86)	0.024^***^ (3.61)	0.019^***^ (3.31)	0.015^***^ (3.80)
Growth	0.027 (1.00)	0.009 (0.38)	0.031^*^ (1.77)	0.077^***^ (2.61)	0.008 (0.30)	0.065^***^ (3.07)
GPDg	0.193 (1.06)	0.366^**^ (2.41)	0.155 (1.33)	0.340^**^ (2.04)	0.499^*^ (1.93)	0.112 (0.90)
_cons	1.249^***^ (7.22)	1.454^***^ (10.53)	1.245^***^ (10.74)	1.216^***^ (5.91)	1.084^***^ (5.30)	1.241^***^ (9.15)
*N*	1,346	1931	2,838	789	978	1917
Year	Y	Y	Y	Y	Y	Y
Industry	Y	Y	Y	Y	Y	Y
Province	Y	Y	Y	Y	Y	Y
Adjust *R*^2^	0.442	0.431	0.445	0.456	0.405	0.448
**Panel B: Negative attitude and corporate cash holdings**						
Supply1	−0.038 (−0.34)					
Demand1		0.020 (0.27)				
P&O1			0.046 (0.84)			
Finance1				−0.196 (−1.46)		
Cost1					0.045 (0.39)	
GOV1						−0.039 (−0.33)
Size	−0.005 (−0.49)	−0.010 (−0.96)	−0.010 (−1.01)	−0.010 (−0.94)	0.001 (0.13)	−0.014
						(−1.32)
Lev	−0.827^***^ (−10.35)	−0.725^***^ (−9.76)	−0.783^***^ (−11.01)	−0.923^***^ (−8.46)	−0.694^***^ (−8.59)	−0.798^***^ (−9.79)
Cashflow	0.244 (1.47)	0.303^*^ (1.91)	0.054 (0.34)	−0.143 (−0.65)	−0.000 (−0.00)	0.159 (0.86)
NWC	−0.432^***^ (−5.35)	−0.319^***^ (−4.43)	−0.340^***^ (−4.84)	−0.500^***^ (−4.56)	−0.395^***^ (−5.38)	−0.357^***^ (−4.59)
Bankdebt	−0.325^***^ (−5.95)	−0.289^***^ (−6.51)	−0.260^***^ (−5.23)	−0.281^***^ (−4.88)	−0.274^***^ (−4.59)	−0.322^***^ (−6.13)
Capex	−1.513^***^ (−5.44)	−1.034^***^ (−4.41)	−1.352^***^ (−5.78)	−1.100^***^ (−3.34)	−1.094^***^ (−3.50)	−1.287^***^ (−4.77)
Dividend	0.049^*^ (1.96)	0.079^***^ (3.53)	0.094^***^ (4.01)	0.083^***^ (2.96)	0.051^**^ (1.98)	0.092^***^ (3.76)
Soe	0.039 (1.30)	0.036 (1.43)	0.047^**^ (2.02)	0.018 (0.72)	0.040 (1.46)	0.021 (0.85)
Board	0.010 (1.39)	−0.000 (−0.02)	0.009 (1.39)	0.010 (1.24)	0.004 (0.57)	0.013^**^ (2.01)
Age	−0.158^***^ (−4.24)	−0.146^***^ (−4.53)	−0.216^***^ (−5.26)	−0.189^***^ (−3.96)	−0.148^***^ (−3.14)	−0.196^***^ (−4.79)
Tobinq	0.035^***^ (3.69)	0.033^***^ (3.52)	0.034^***^ (3.56)	0.017 (1.42)	0.042^***^ (3.96)	0.030^***^ (3.14)
Growth	0.015 (0.42)	0.022 (0.56)	0.027 (0.85)	0.031 (0.79)	0.047 (1.33)	−0.031 (−0.77)
GPDg	−0.224 (−0.71)	0.116 (0.60)	0.212 (1.14)	0.164 (0.57)	0.563 (1.57)	0.250 (0.91)
_cons	1.296^***^ (4.41)	1.276^***^ (4.81)	1.443^***^ (5.65)	1.562^***^ (4.73)	1.068^***^ (3.90)	1.541^***^ (5.95)
*N*	643	786	885	530	529	715
Year	Y	Y	Y	Y	Y	Y
Industry	Y	Y	Y	Y	Y	Y
Province	Y	Y	Y	Y	Y	Y
Adjust *R*^2^	0.522	0.501	0.504	0.497	0.520	0.502

***, **, and * indicate statistical significance at the 1%, 5%, and 10% levels, respectively; the *t* value is in brackets; the regression in this table is clustered according to the company code.

Table 11 shows the regression results of the six major topics of the COVID-19 pandemic and corporate cash holdings in the future outlook. Panel A in Table 11 shows that when the management tone is positive, these six major topics have no significant relationship with the company’s future cash holdings. Panel B in Table 11 shows that when the management tone is negative, the regression coefficients of supply chain (Supply2), demand (Demand2), production and operation (P&O2) government (GOV2) are significantly negative, indicating that the impact of the epidemic on the supply chain, demand, production operations and the implementation of relevant government policies may has severely affected corporate performance, consist with the findings in Table 3, resulting in a shortage of cash flow and hence a decrease of cash holding levels of companies in the future.

**Table 11:** Six major topics of COVID-19 and corporate cash holdings (future outlook section).

	(1)	(2)	(3)	(4)	(5)	(6)
	Cash*_t_*	Cash*_t_*	Cash*_t_*	Cash*_t_*	Cash*_t_*	Cash*_t_*
**Panel A: Positive attitude and corporate cash holdings**						
Supply2	0.004 (0.02)					
Demand2		−0.042 (−0.44)				
P&O2			0.025 (0.39)			
Finance2				0.037 (0.09)		
Cost2					−0.027 (−0.13)	
GOV2						0.100 (1.15)
Size	−0.012 (−1.26)	−0.007 (−0.82)	−0.008 (−1.09)	−0.012 (−0.69)	−0.007 (−0.46)	−0.008 (−1.04)
Lev	−0.614^***^ (−6.23)	−0.597^***^ (−8.06)	−0.582^***^ (−9.50)	−0.699^***^ (−4.86)	−0.619^***^ (−4.62)	−0.562^***^ (−7.79)
Cashflow	0.204 (0.97)	0.404^***^ (2.64)	0.476^***^ (3.66)	−0.198 (−0.67)	0.651^**^ (2.17)	0.377^**^ (2.50)
NWC	−0.394^***^ (−4.97)	−0.377^***^ (−6.03)	−0.402^***^ (−7.19)	−0.565^***^ (−5.00)	−0.469^***^ (−4.48)	−0.375^***^ (−6.26)
Bankdebt	−0.413^***^ (−6.50)	−0.327^***^ (−6.92)	−0.309^***^ (−7.57)	−0.397^***^ (−4.36)	−0.352^***^ (−4.71)	−0.357^***^ (−7.43)
Capex	−0.347 (−1.13)	−0.427^*^ (−1.81)	−0.762^***^ (−3.76)	−1.147^**^ (−2.34)	−0.365 (−0.95)	−0.593^**^ (−2.51)
Dividend	0.017 (0.77)	0.014 (0.74)	0.033^**^ (2.22)	0.026 (0.84)	0.052^*^ (1.67)	0.018 (0.94)
Soe	0.034 (1.09)	0.022 (1.06)	0.024 (1.33)	−0.003 (−0.06)	−0.004 (−0.11)	0.019 (0.91)
Board	0.020^**^ (2.47)	0.003 (0.50)	0.002 (0.31)	0.029^**^ (2.38)	0.018 (1.58)	0.003 (0.50)
Age	−0.144^***^ (−3.18)	−0.189^***^ (−3.97)	−0.133^***^ (−3.34)	−0.099 (−1.39)	−0.071 (−0.93)	−0.075^*^ (−1.78)
Tobinq	0.000 (0.02)	0.006 (0.73)	0.007 (1.01)	0.004 (0.27)	0.000 (0.01)	0.001 (0.10)
Growth	−0.028 (−0.64)	0.026 (0.74)	0.009 (0.32)	−0.042 (−0.81)	−0.019 (−0.33)	0.009 (0.29)
GPDg	0.516^*^ (1.79)	0.993^***^ (3.32)	0.599^**^ (2.18)	0.901^*^ (1.78)	0.454 (1.12)	0.269 (0.84)
_cons	1.128^***^ (4.07)	1.341^***^ (5.30)	1.204^***^ (5.66)	1.075^**^ (2.56)	0.854^**^ (2.25)	0.956^***^ (4.14)
*N*	434	673	898	243	281	672
Year	Y	Y	Y	Y	Y	Y
Industry	Y	Y	Y	Y	Y	Y
Province	Y	Y	Y	Y	Y	Y
Adjust *R*^2^	0.365	0.367	0.381	0.379	0.389	0.330
**Panel B: Negative attitude and corporate cash holdings**						
Supply2	−0.323^***^ (−2.62)					
Demand2		−0.172^*^ (−1.66)				
P&O2			−0.149^**^ (−1.99)			
Finance2				0.123 (0.84)		
Cost2					0.019 (0.07)	
GOV2						−0.234^**^ (−2.18)
Size	−0.008 (−0.69)	−0.005 (−0.54)	−0.001 (−0.06)	0.010 (0.81)	0.008 (0.54)	0.003 (0.28)
Lev	−0.588^***^ (−5.88)	−0.518^***^ (−5.77)	−0.559^***^ (−7.40)	−0.737^***^ (−6.73)	−0.810^***^ (−5.89)	−0.565^***^ (−6.00)
Cashflow	0.396^*^ (1.94)	0.124 (0.62)	0.126 (0.82)	0.338^*^ (1.66)	0.167 (0.63)	0.182 (0.97)
NWC	−0.299^***^ (−4.65)	−0.338^***^ (−5.22)	−0.318^***^ (−5.66)	−0.272^***^ (−4.21)	−0.526^***^ (−5.57)	−0.393^***^ (−5.07)
Bankdebt	−0.222^***^ (−3.54)	−0.269^***^ (−4.45)	−0.263^***^ (−5.44)	−0.190^**^ (−2.21)	−0.383^***^ (−4.79)	−0.280^***^ (−4.93)
Capex	−0.359 (−1.31)	−0.839^***^ (−3.24)	−0.762^***^ (−3.48)	−0.536 (−1.25)	−0.352 (−0.95)	−0.689^**^ (−2.53)
Dividend	0.027 (1.17)	0.061^***^ (2.62)	0.049^***^ (2.68)	0.047^*^ (1.74)	0.063^*^ (1.89)	0.058^***^ (2.79)
Soe	0.030 (1.05)	−0.008 (−0.26)	0.013 (0.58)	0.101^***^ (3.82)	−0.029 (−0.81)	0.006 (0.25)
Board	0.000 (0.03)	−0.002 (−0.26)	−0.006 (−0.99)	−0.002 (−0.27)	0.007 (0.81)	0.001 (0.20)
Age	−0.117^**^ (−2.21)	−0.061 (−1.38)	−0.056 (−1.48)	−0.169^***^ (−2.72)	−0.140^**^ (−2.19)	−0.127^***^ (−2.81)
Tobinq	0.012 (1.46)	0.006 (0.85)	0.013^*^ (1.72)	0.011 (1.21)	−0.001 (−0.10)	0.007 (0.65)
Growth	−0.076 (−1.61)	−0.045 (−1.09)	−0.024 (−0.75)	−0.046 (−1.06)	−0.079 (−1.37)	0.004 (0.10)
GPDg	−0.186 (−0.34)	0.619 (1.37)	0.402 (1.19)	0.136 (0.18)	1.591^***^ (3.27)	0.189 (0.37)
_cons	1.024^***^ (3.26)	0.942^***^ (3.38)	0.765^***^ (3.03)	0.984^***^ (3.43)	0.991^***^ (2.69)	0.775^**^ (2.57)
*N*	406	433	647	338	242	469
Year	Y	Y	Y	Y	Y	Y
Industry	Y	Y	Y	Y	Y	Y
Province	Y	Y	Y	Y	Y	Y
Adjust *R*^2^	0.458	0.379	0.389	0.444	0.486	0.394

***, **, and * indicate statistical significance at the 1%, 5%, and 10% levels, respectively; the *t* value is in brackets; the regression in this table is clustered according to the company code.

#### COVID-19 impact and corporate cash holdings: Based on the type of ownership

The ownership nature of a business may play a different role in the impact of cash holdings levels. Referring to Liu et al. (2015), we focus on whether the previous findings are mainly due to SOEs or non-SOEs. Specifically, we divide the sample into state-owned (Soe = 1) and non-state-owned (Soe = 0) companies to complete a regression model. The results in Table 12 show that the positive relationship between the impact of the pandemic and the current cash holdings levels of enterprises is only significant in the group of state-owned enterprises (Soe = 1), and the negative relationship with the future cash holdings of enterprises is only significant in non-state-owned enterprises (Soe = 0). The reasons we perceive in this study are that in the face of the impact of the COVID-19 epidemic, state-owned enterprises are more vulnerable to the impact of the government’s relevant epidemic prevention policies and more sensitive to the impact of the pandemic; at the same time, state-owned enterprises are also more likely to obtain more free cash in the event of a credit crisis. As a consequence of this cash injection, the impact of the COVID-19 pandemic on its current cash holdings have increased significantly. In contrast, non-state-owned enterprises may have their cash holdings significantly reduced due to financing constraints and their reduced performance.

**Table 12:** COVID-19 impact and corporate cash holdings: based on the type of ownership.

	Cash*_t_*		Cash*_t + 1_*	
	(1)	(2)	(3)	(4)
	Soe = 0	Soe = 1	Soe = 0	Soe = 1
Covid1	0.073 (1.53)	0.116^**^ (1.98)		
Covid2			−0.145^**^ (−2.47)	−0.107 (−1.31)
Size	−0.007 (−1.47)	−0.008 (−1.46)	−0.004 (−0.96)	−0.007 (−1.22)
Lev	−0.775^***^ (−20.15)	−0.416^***^ (−9.24)	−0.714^***^ (−16.99)	−0.421^***^ (−8.53)
Cashflow	0.124^*^ (1.87)	0.292^***^ (3.36)	0.178^**^ (2.28)	0.300^**^ (2.47)
NWC	−0.417^***^ (−12.86)	−0.367^***^ (−8.87)	−0.427^***^ (−12.24)	−0.368^***^ (−8.57)
Bankdebt	−0.328^***^ (−15.05)	−0.259^***^ (−8.99)	−0.299^***^ (−12.06)	−0.260^***^ (−8.22)
Capex	−1.008^***^ (−10.51)	−1.269^***^ (−8.20)	−0.762^***^ (−7.02)	−1.165^***^ (−6.33)
Dividend	0.068^***^ (7.94)	0.037^***^ (3.63)	0.054^***^ (5.36)	0.024^**^ (1.96)
Board	0.004 (1.42)	−0.001 (−0.16)	0.002 (0.57)	−0.001 (−0.18)
Age	−0.164^***^ (−9.61)	−0.048^*^ (−1.84)	−0.130^***^ (−6.69)	−0.031 (−1.19)
Tobinq	0.021^***^ (6.68)	0.030^***^ (3.99)	0.012^***^ (3.81)	0.022^***^ (2.94)
Growth	0.027^**^ (2.03)	−0.009 (−0.47)	0.020 (1.15)	−0.017 (−0.59)
GDPg	0.066 (1.04)	0.149^***^ (2.60)	0.427^**^ (2.28)	0.485^*^ (1.86)
_cons	1.276^***^ (10.77)	0.796^***^ (5.09)	1.113^***^ (9.33)	0.760^***^ (4.64)
*N*	4,978	2,155	2,260	1,067
Year	Y	Y	Y	Y
Industry	Y	Y	Y	Y
Province	Y	Y	Y	Y
Adjust *R*^2^	0.432	0.452	0.427	0.428

***, **, and * indicate statistical significance at the 1%, 5%, and 10% levels, respectively; the t value is in brackets; the regression in this table is clustered according to the company code.

#### COVID-19 impact and corporate cash holdings: Based on population density

At the same time, the social environment in which the company operates may have an effect on the relationship between the impact of the pandemic and its cash holdings. Therefore, based on the median of population density (Population), this paper divides the samples into two groups: high population density (H-Population) and low population density (L-Population). The regression was completed in this basis with the outcomes shown in Table 13. The results show that the positive relationship between the impact of the pandemic and the company’s current cash holdings and the negative relationship with the company’s future cash holdings are only significant in groups with high population density. Due to the COVID-19 characteristics of human-to-human transmission, the higher the population density of the province where the company is located, the more sensitive it may be to the impact of the pandemic. This means that companies are either more likely to increase the level of cash holdings in the current period or its cash holdings level will drop significantly due to the major impact on the company’s performance.

**Table 13:** COVID-19 impact and corporate cash holdings: based on population density.

	Cash*_t_*		Cash_*t* + 1_	
	(1)	(2)	(3)	(4)
	H-Population	L-Population	H-Population	L-Population
Covid1	0.102^*^ (1.71)	0.061 (1.24)		
Covid2			−0.168^**^ (−2.38)	−0.088 (−1.33)
Size	−0.007 (−1.29)	−0.011^**^ (−2.10)	−0.011^**^ (−2.10)	−0.005 (−0.90)
Lev	−0.710^***^ (−16.87)	−0.573^***^ (−14.00)	−0.600^***^ (−12.79)	−0.587^***^ (−13.16)
Cashflow	0.130^*^ (1.67)	0.239^***^ (3.31)	0.343^***^ (3.50)	0.100 (1.16)
NWC	−0.388^***^ (−10.54)	−0.375^***^ (−10.26)	−0.390^***^ (−9.84)	−0.397^***^ (−10.22)
Bankdebt	−0.304^***^ (−11.89)	−0.317^***^ (−13.41)	−0.293^***^ (−9.97)	−0.292^***^ (−11.19)
Capex	−1.241^***^ (−10.16)	−0.888^***^ (−8.26)	−0.896^***^ (−6.14)	−0.828^***^ (−6.83)
Dividend	0.079^***^ (8.14)	0.043^***^ (4.74)	0.057^***^ (4.78)	0.031^***^ (2.95)
Soe	0.015 (1.15)	0.014 (1.29)	0.021 (1.51)	0.013 (1.10)
Board	0.004 (1.18)	0.001 (0.45)	−0.000 (−0.08)	0.002 (0.83)
Age	−0.165^***^ (−8.79)	−0.110^***^ (−5.09)	−0.123^***^ (−5.66)	−0.083^***^ (−3.69)
Tobinq	0.029^***^ (6.45)	0.020^***^ (5.76)	0.017^***^ (3.74)	0.015^***^ (4.08)
Growth	0.021 (1.33)	0.012 (0.74)	−0.003 (−0.11)	0.029 (1.35)
GPDg	0.065 (1.02)	0.173^***^ (2.60)	0.058 (0.16)	0.472^***^ (3.05)
_cons	1.202^***^ (9.45)	1.087^***^ (6.48)	1.185^***^ (8.58)	0.906^***^ (5.05)
*N*	3,772	3,361	1,671	1,656
Year	Y	Y	Y	Y
Industry	Y	Y	Y	Y
Province	Y	Y	Y	Y
Adjust *R*^2^	0.435	0.413	0.415	0.407

***, **, and * indicate statistical significance at the 1%, 5%, and 10% levels, respectively; the *t* value is in brackets; the regression in this table is clustered according to the company code.

#### COVID-19 impact and corporate cash holdings: Based on industry competition

In addition, the industry environment in which the company operates may also have an impact on the relationship between the impact of the COVID-19 pandemic and corporate cash holdings (Yang et al., 2016). Therefore, this paper further uses the median of the market concentration degree (HHI) as the grouping basis and also divides the sample into two subsamples of high market concentration (H-HHI) and low market concentration (L-HHI) to run the regression model. Results are shown in Table 14 and show that the positive relationship between the impact of the pandemic and the company’s current cash holdings and the negative relationship with the company’s future cash holdings are only significant in the group with low market concentration area, i.e., high degree of industry competition area. The possible reason is that in the face of exogenous impact, the higher the degree of industry competition, the higher the sensitivity of the industry to risks and the easier it is to respond significantly to the impact of the pandemic.

**Table 14:** COVID-19 impact and corporate cash holdings: based on industry competition.

	Cash*_t_*		Cash*_t + 1_*	
	(1)	(2)	(3)	(4)
	H-HHI	L-HHI	H-HHI	L-HHI
Covid1	0.036 (0.60)	0.114^**^ (2.32)		
Covid2			−0.071 (−1.00)	−0.190^***^ (−2.89)
Size	−0.010^*^ (−1.84)	−0.009^*^ (−1.84)	−0.009 (−1.50)	−0.007 (−1.61)
Lev	−0.665^***^ (−15.55)	−0.640^***^ (−15.41)	−0.611^***^ (−12.76)	−0.591^***^ (−13.73)
Cashflow	0.191^**^ (2.44)	0.149^**^ (2.02)	0.201^**^ (2.09)	0.223^***^ (2.63)
NWC	−0.393^***^ (−10.25)	−0.394^***^ (−11.28)	−0.423^***^ (−10.16)	−0.375^***^ (−10.49)
Bankdebt	−0.315^***^ (−12.15)	−0.299^***^ (−12.86)	−0.318^***^ (−10.58)	−0.263^***^ (−10.52)
Capex	−1.290^***^ (−10.70)	−0.852^***^ (−7.87)	−1.157^***^ (−8.41)	−0.526^***^ (−4.14)
Dividend	0.070^***^ (6.88)	0.054^***^ (6.21)	0.051^***^ (4.12)	0.037^***^ (3.72)
Soe	0.012 (0.90)	0.014 (1.28)	0.010 (0.73)	0.017 (1.51)
Board	0.006^*^ (1.65)	−0.000 (−0.05)	0.004 (1.19)	−0.002 (−0.71)
Age	−0.141^***^ (−7.18)	−0.141^***^ (−6.72)	−0.118^***^ (−5.24)	−0.078^***^ (−3.57)
Tobinq	0.028^***^ (6.76)	0.021^***^ (5.24)	0.021^***^ (4.65)	0.011^***^ (2.81)
Growth	0.007 (0.42)	0.031^**^ (2.06)	−0.002 (−0.09)	0.024 (1.17)
GDPg	0.101 (1.58)	0.091 (1.48)	0.529^**^ (2.34)	0.343^*^ (1.88)
_cons	1.195^***^ (8.95)	1.272^***^ (9.73)	1.114^***^ (7.80)	0.975^***^ (7.74)
*N*	3,605	3,528	1,678	1,649
Year	Y	Y	Y	Y
Industry	Y	Y	Y	Y
Province	Y	Y	Y	Y
Adjust *R*^2^	0.424	0.425	0.419	0.408

***, **, and * indicate statistical significance at the 1%, 5%, and 10% levels, respectively; the *t* value is in brackets; the regression in this table is clustered according to the company code.

## Conclusion and implication

This paper discusses the relationship between the impact of the COVID-19 pandemic and corporate cash holdings levels. The study finds that the impact of the COVID-19 outbreak leads to an increase in current cash holdings and a decrease in future cash holdings. At the same time, based on the theory of precautionary motive, the more positive the management team’s attitude towards the pandemic, the lower the current cash holdings level of the company. On the contrary, the more negative the management team’s attitude towards the pandemic, the higher the current cash holdings level of the company. In addition, the confidence of the management team in the supply chain and the government policies has reduced the company’s current precautionary motive to a certain extent. We also found that the impact of the COVID-19 pandemic on the supply chain, demand, production operations and the implementation of relevant government policies is likely to have a negative impact on the company’s performance. Which result in a decrease in the level of future cash holdings of the company. Furthermore, the impact of the pandemic on the cash holdings level of state-owned enterprises is mainly reflected in the current period, whereas the impact on non-state-owned enterprises is mainly reflected in the future. Last but not least, the greater the population density and the more intense the industry competition, the more significant the impact of the pandemic on the current and future cash holdings of companies.

We trust that the conclusions of this paper have a theoretical significance and practical value. The impact of the COVID-19 pandemic has caused serious disruptions to the internal economic cycle not only in China but also in other countries, and the ability to unblock the internal economic cycle of enterprises is a key point to promote the recovery of the economic cycle in all countries. The conclusion of this paper shows that the impact of the COVID-19 pandemic has had a great impact on the level of corporate cash holdings. Enterprises and the government should take prompt measures to accelerate the recovery of the domestic and international economic cycle. Specifically, the enterprises should be fully aware of the importance of maintaining an appropriate level of cash flow, actively strengthen the management of their cash holdings, and accelerate the flow of internal capital circulation in order to promote economic recovery and enhance their ability to cope with exogenous shocks. And the government should promote the economic cycle recovery from both supply and demand sides. On the supply-side, effective measures are needed to prevent and control the epidemic, and thus making timely and orderly arrangements to resume work and production, and promoting the steady recovery of enterprises’ production and operation, so as to alleviate the difficulties faced by enterprises in the supply chain as well as production and operation. On the demand-side, it can steadily promote the rebound of consumers’ demand with the help of policies such as consumer subsidies, tax and fee reductions.

Of course, we are aware that this paper examines the important role of firms’ internal cash level management (including investment and financing decisions) in coping with epidemic shocks and does not provide an in-depth exploration of how external governance plays a role in firms’ response to uncertainty shocks. Future research may be interesting to cut through the lens of external governance (e.g., institutional investors) to explore whether and to what extent external governance can help mitigate the risks associated with the great external uncertainties that firms may face, thus providing further empirical evidence for smooth corporate operations and stable global economic development.

## Data availability statement

The original contributions presented in the study are included in the article/Supplementary material, further inquiries can be directed to the corresponding author/s.

## Author contributions

DZ contributed to conception and design of the study. HZ organized the database. MB performed the statistical analysis. YQ wrote the first draft of the manuscript. All authors contributed to the article and approved the submitted version.

## Funding

This study was funded by the National Natural Science Foundation of China (No. 71972091).

## Conflict of interest

The authors declare that the research was conducted in the absence of any commercial or financial relationships that could be construed as a potential conflict of interest.

## Publisher’s note

All claims expressed in this article are solely those of the authors and do not necessarily represent those of their affiliated organizations, or those of the publisher, the editors and the reviewers. Any product that may be evaluated in this article, or claim that may be made by its manufacturer, is not guaranteed or endorsed by the publisher.

## Footnotes

^1^3.05% = 3.542 × 0.068/7.894.
